# Immune regulation and cell metabolism in B cell subsets in patients with systemic lupus erythematosus

**DOI:** 10.3389/fimmu.2026.1755589

**Published:** 2026-05-26

**Authors:** Masanobu Ueno, Satoshi Kubo, Yasuyuki Todoroki, Shingo Nakayamada, Yoshiya Tanaka

**Affiliations:** 1 The First Department of Internal Medicine, School of Medicine, University of Occupational and Environmental Health, Kitakyushu, Japan; 2 Department of Molecular Targeted Therapeutics, University of Occupational and Environmental Health, Kitakyushu, Japan

**Keywords:** B cell, glycolysis, immunometabolism, OXPHOS, systemic lupus eryhematosus

## Abstract

Systemic lupus erythematosus (SLE) is an autoimmune disease that predominantly affects young women and involves multiple organs, including the skin, joints, kidneys, and nervous system. In the pathogenesis of SLE, autoreactive B cells play a pivotal role through mediating antibody production, antigen presentation, and cytokine secretion. Recent studies have highlighted immunometabolism as a pivotal regulatory axis operating at both intracellular and extracellular levels during immune dysregulation. In SLE, plasma cells produce large quantities of autoantibodies, playing an important role in disease progression. This process demands a substantial energy supply, along with protein and nucleic acid synthesis, and is accompanied by dynamic reconstitution of intracellular metabolism. Furthermore, alterations in metabolic pathways not only facilitate efficient energy production but also directly modulate immune responses, including cytokine production and cell differentiation. Differences in cellular metabolism can shape B cell differentiation trajectories. Elucidating the immunometabolic mechanisms governing B cell activation and fate decisions may reveal novel drivers of lupus pathogenesis and identify new opportunities for therapeutic intervention.

## Introduction

Systemic lupus erythematosus (SLE) is a chronic autoimmune disease that predominantly affects young women. It often involves multiple organs, including the skin, joints, kidneys, nervous system, and hematopoietic system. Although various immune cell types contribute to lupus pathogenesis, B cells play a central role through their functions in antibody production, cytokine secretion, and antigen presentation (1, 2). Advances in molecular targeted therapies and cell-based therapies directed at B cells have led to substantial progress in the treatment of SLE. However, treatment-refractory cases still exist, underscoring the need for identifying novel biomarkers and therapeutic targets.

Against this background, immunometabolism, a fundamental mechanism regulating immune cell activation and differentiation, has emerged as a new perspective for gaining deeper insight into the molecular basis of disease pathogenesis. The relevance of immunometabolism to the pathogenesis of autoimmune diseases is also becoming increasingly evident. In particular, because B cells exhibit high metabolic demand, dysregulated metabolic control is suggested to play a critical role in disease pathogenesis.

In this review, we focus on immunometabolic abnormalities in B cells in SLE. We summarize recent advances in our understanding of how metabolic regulation influences B-cell activation, differentiation, and disease pathogenesis and discuss the potential implications for future therapeutic applications.

## Disruption of B-cell tolerance and pathogenesis in SLE

In healthy individuals, multiple central and peripheral tolerance checkpoints operate sequentially during B-cell differentiation. At early stages of B-cell development in the bone marrow, V(D)J recombination frequently generates autoreactive B cells; however, these cells are stringently controlled through B-cell receptor (BCR) editing, clonal deletion, or induction of anergy. Furthermore, additional tolerance checkpoints operate during the differentiation of transitional B cells that have emigrated from the bone marrow into mature naïve B cells. Through these mechanisms, autoreactive clones are further eliminated or functionally silenced (3–5). Therefore, self-tolerance does not rely solely on peripheral differentiation. Instead, it is established in a stepwise and multilayered manner through multiple independent checkpoints, spanning early B-cell development in the bone marrow to peripheral maturation.

In SLE, these tolerance mechanisms are disrupted, and abnormalities have been identified at multiple checkpoints, ranging from central B-cell tolerance in the bone marrow to peripheral maturation. In patients with SLE, autoreactive B cells are already significantly increased at the transitional and mature naïve B-cell stages. This finding indicates that B-cell tolerance is breached at early stages prior to antigen stimulation or germinal center reactions (4–7). Under conditions in which autoreactive B cells persist and accumulate within the immune repertoire, additional signals such as BCR cross-linking; CD40 stimulation mediated by soluble CD40 ligand (sCD40L); Toll-like receptor (TLR) signaling; and inflammatory cytokines such as IFN-α and IL-21 promote B-cell activation, class-switch recombination, differentiation into plasma cells, and excessive autoantibody production (8–10). These signaling pathways are not specific to autoreactive B cells but represent general activation pathways that can act on all B cells. However, in SLE, the coexistence of an immune environment enriched in autoreactive B cells and a chronic inflammatory milieu dominated by type I interferon is thought to render these physiological activation signals pathogenic, resulting in aberrant B-cell responses (11). These autoantibodies generated through the above processes form immune complexes with endogenous nucleic acids, are deposited in tissues, and induce tissue damage via complement activation. B cells can also act as antigen-presenting cells, presenting self-antigens to T cells and thereby enhancing the production of inflammatory cytokines (12). Consistent with these observations, Our group has previously reported on the pathogenic role of B cells in SLE (13–17).

## B cell targeted therapies in SLE: current status and challenges

The introduction of glucocorticoids (GCs) has markedly improved the prognosis of SLE. However, current GC-based therapies still have important limitations, including an increased risk of infections, higher mortality due to cardiovascular events, and reduced activities of daily living caused by complications such as diabetes mellitus and osteoporosis (18, 19). Against this background, there has been strong demand for the development of therapeutic strategies to reduce dependence on GCs.

In recent years, the development of molecular targeted therapies for SLE has advanced, and disease control strategies centered on B cells have attracted increasing attention. Belimumab, a monoclonal antibody against B-cell activating factor (BAFF), modulates B-cell survival and differentiation by inhibiting BAFF signaling and has been approved for the treatment of refractory SLE (20). Furthermore, in August 2023, the anti-CD20 antibody rituximab, which suppresses pathogenic B-cell responses through direct depletion of CD20-positive B cells, was approved for reimbursement for lupus nephritis for the first time only in Japan, thereby expanding therapeutic options. These B-cell-targeted therapies have demonstrated clinical benefits, including disease activity control and GC-sparing effects, in real-world practice (21–24).

Chimeric antigen receptor-modified T-cell (CAR-T) therapy targeting B-cell antigens has been used to treat SLE more recently, with successful cases of CD19-targeted CAR-T therapy having been reported (25, 26). Although the expansion of therapeutic options has improved the clinical outcomes in SLE, a subset of patients remains refractory to treatment. Therefore, elucidating the mechanisms underlying the pathogenesis of SLE and identifying novel biomarkers and therapeutic targets remain pressing priorities. Increasing attention has been paid in recent years to immunometabolism that regulates the activation and differentiation of immune cells. Immunometabolism is a concept that integrates the regulation of cytokine production and cell differentiation through intracellular energy production and biosynthesis and has been shown to play a crucial role in the pathogenesis of autoimmune diseases (27–29). Because B cells produce large amounts of autoantibodies, they require substantial energy, as well as nutrients for protein and nucleic acid synthesis. Metabolic abnormalities in B cells have been reported in patients with SLE (29). These findings suggest that intracellular metabolic pathways may underlie aberrant B cell activation and excessive antibody production in SLE. This review summarizes recent advances in our understanding of the mechanisms underlying B cell activation through immunometabolic regulation and their implications in SLE pathogenesis.

### Metabolic regulation of B cells

**Figure 1:**
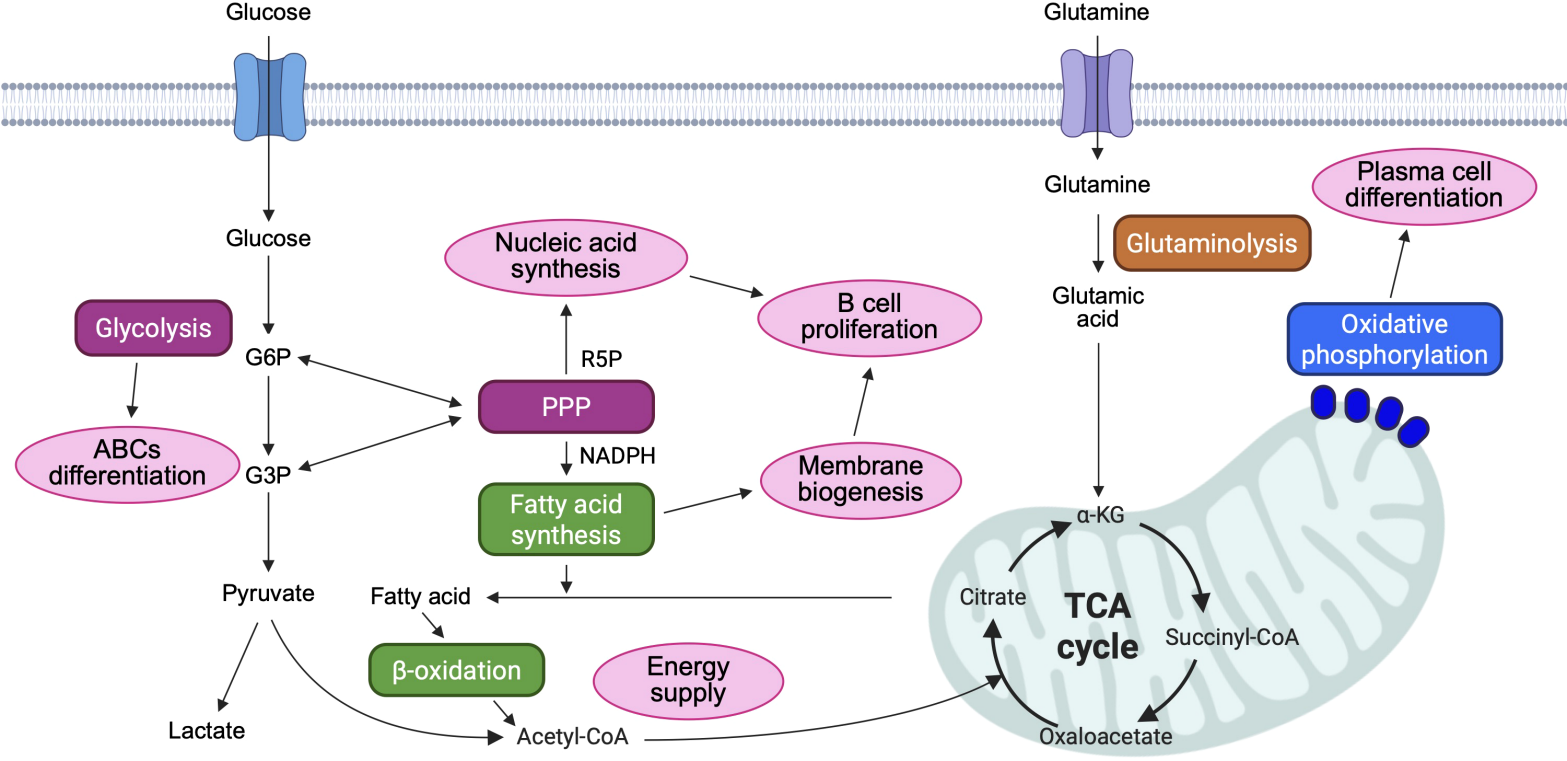
Cellular metabolic pathway. There are six major intracellular metabolic pathways: glycolysis, the pentose phosphate pathway, oxidative phosphorylation, fatty acid oxidation (β-oxidation), fatty acid synthesis, and amino acid metabolism (glutaminolysis). G6P; glucose-6-phosphate, G3P; glyceraldehyde-3-phosphate, R5P; ribose-5-phosphate, PPP; pentose phosphate pathway, NADPH; nicotinamide adenine dinucleotide phosphate, α-KG; α-ketoglutarate, TCA; tricarboxylic acid cycle.

The differentiation, proliferation, and activation of immune cells require substantial energy production and synthesis of cellular components. These processes are supported by intracellular metabolism, which consists of six major pathways: (1) glycolysis, (2) the pentose phosphate pathway, (3) oxidative phosphorylation (OXPHOS), (4) fatty acid oxidation (β-oxidation), (5) fatty acid synthesis, and (6) amino acid metabolism. These metabolic pathways do not exist independently, being interconnected and mutually compensatory. Alterations in their balance have been shown to closely influence the activation states and differentiation pathways of immune cells (**Figure 1**) (30, 31).

Glycolysis is a metabolic pathway that generates ATP independently of oxygen availability and is regarded as a central hub of intracellular metabolism, enabling rapid energy supply. Although the efficiency of ATP production is lower than that of oxidative phosphorylation (OXPHOS), glycolysis can provide ATP rapidly. In addition, glycolysis promotes the production of NADPH and ribose-5-phosphate by supplying glucose-6-phosphate to the pentose phosphate pathway. These metabolic products support biosynthetic processes essential for cell proliferation, including lipid and nucleotide synthesis. Based on these properties, conditions requiring rapid ATP supply, such as cellular activation and proliferation, are associated with a metabolic shift toward glycolysis even in the presence of sufficient oxygen. This phenomenon is known as the Warburg effect or aerobic glycolysis (32). This metabolic shift is important not only for rapid ATP production but also because it promotes mitochondrial maturation and enhances the tricarboxylic acid (TCA) cycle and OXPHOS (30, 33, 34). Furthermore, the TCA cycle and OXPHOS represent major intracellular metabolic hubs alongside glycolysis and are interconnected through amino acid and lipid metabolism. Following mitochondrial maturation, these metabolic pathways become activated, enabling efficient ATP production predominantly through OXPHOS. Glycogen synthase kinase 3, conversely, suppresses cell proliferation and metabolic activity in resting immune cells, thereby maintaining redox homeostasis and supporting a long-term quiescent state (35).

These metabolic pathways play functionally important roles in B cells. The pentose phosphate pathway supports antioxidant responses and nucleotide biosynthesis by supplying NADPH and ribose-5-phosphate. These functions contribute to the proliferation of germinal center B cells and their differentiation into antibody-producing cells (36). Lipid metabolism is also important for B cell differentiation. Fatty acid synthesis promotes membrane biogenesis and endoplasmic reticulum expansion, thereby facilitating the intracellular structural remodeling required for efficient antibody secretion. In particular, ACLY-dependent *de novo* fatty acid synthesis is essential for plasma cell differentiation and the maintenance of immunoglobulin production (37). In addition to its role in energy supply, lipid metabolism has been implicated in the regulation of B cell differentiation through signaling pathways. Short-chain fatty acids (SCFAs) derived from the gut microbiota have been reported to suppress NF-κB activity and PRDM1 expression via GPR43-mediated signaling, thereby inhibiting plasma cell differentiation and antibody production (38). Furthermore, amino acid metabolism plays a role in the regulation of B cell differentiation. In B cells, branched-chain amino acid transaminase 1 (BCAT1) is induced upon BCR and TLR9 stimulation, and regulates cellular proliferation and functional responses through activation of mTORC1 signaling (39).

Metabolic reprogramming of B cells in response to activation signals also provides a critical foundation for proliferation, differentiation, and functional responses. Upon BCR stimulation, activation of Syk drives the downstream PI3K-Akt-mTORC1 pathway, resulting in enhanced glycolysis and oxidative phosphorylation (40). Similarly, stimulation through TLR9 or type I interferons induces mTORC1 activation in B cells, highlighting the close interplay between innate immune signaling and metabolic regulation (41). Upon B-cell activation, glycolysis and OXPHOS are coordinately increased. However, the inhibition of glycolysis or a Glut1 deficiency reduces antibody production, which indicates that glucose metabolism is essential for B-cell function (42). In addition, hexokinase 2 (HK2), which is induced in a PI3K-dependent manner, plays an important role in B-cell responses. This finding suggests that metabolic enzymes can directly regulate B-cell function (43). MCT1 regulates B-cell activation through pyruvate metabolism and is involved in class-switch recombination and antibody production. In particular, MCT1-dependent pyruvate metabolism supports the supply of acetyl-CoA required for H3K27 acetylation. Through this mechanism, MCT1 has been shown to promote the expression of genes required for class-switch recombination via epigenetic regulation (44). In contrast, during the early phase of B-cell activation, glycolysis has a limited contribution as glucose is mainly used for nucleotide biosynthesis, whereas oxidative phosphorylation and glutamine metabolism are important for cell proliferation and differentiation (36). Furthermore, BCR stimulation-induced metabolic activation is transient. In the absence of co-stimulatory signals, B cells undergo mitochondrial dysfunction followed by apoptosis. This indicates that the metabolic state is closely linked to B-cell survival (45). Taken together, these findings suggest that metabolic activation is not only a source of energy but also a key regulator of B-cell activation and differentiation.

In B cells from patients with SLE, increased expression of glycolysis-related genes and the establishment of a glycolytic state coupled to mTORC1 activity have been reported (46, 47). This enhanced glycolysis is thought to function not only as a source of rapid energy supply but also as a metabolic foundation that promotes mitochondrial activation. Through this mechanism, glycolytic reprogramming supports B-cell activation and differentiation. In SLE B cells, mitochondrial dysfunctions such as hyperpolarization of the mitochondrial membrane potential, increased oxygen consumption rates, and elevated production of reactive oxygen species (ROS) have been observed. These mitochondrial abnormalities have been reported to correlate with increased plasma cell frequencies and disease activity (48). Furthermore, enhanced mitochondrial activity is accompanied by increased amino acid metabolism, which promotes plasma cell differentiation and contributes to SLE pathogenesis (49). Conversely, inhibition of amino acid metabolism reduces mitochondrial membrane hyperpolarization, oxygen consumption rates, and ROS production. This metabolic suppression leads to decreased plasma cell differentiation and antibody production, highlighting the close interdependence between B-cell function and metabolic state (48).

In recent years, the nucleotype pathway has attracted attention as a conceptual framework linking nucleotide metabolism to innate immune responses. DNA and RNA are strongly regulated during their synthesis and degradation and are normally retained within the nucleus and mitochondria. However, when nucleotide metabolic dysregulation or mitochondrial dysfunction disrupts intracellular nucleotide pool homeostasis, mitochondrial DNA can leak into the cytosol. As a consequence, type I interferon responses and inflammatory signaling are induced through activation of the cGAS-STING pathway (50, 51). Indeed, transcriptomic analyses of B cells from patients with SLE have demonstrated that reprogramming of nucleotide metabolism and mitochondrial pathways occurs in close association with a sustained interferon-driven inflammatory state. Together, these findings suggest that exposure of B cells to inflammatory cytokines and type I interferon signaling may drive the acquisition of metabolic programs adapted to a pathological inflammatory environment (52).

### B cell metabolic reprogramming in SLE

The two major subsets involved in the abnormal differentiation of peripheral B cells in SLE are plasma and age-associated B cells (ABCs). B cells are thought to be chronically exposed to stimulatory signals in SLE, with abnormal peripheral B cell differentiation being frequently observed (14). Both of these B-cell subsets are deeply involved in disease pathogenesis. However, recent studies have revealed that the intracellular metabolic programs governing their differentiation and functions are fundamentally distinct.

First, plasma cells are terminally differentiated cells responsible for the production of large amounts of immunoglobulins, and their differentiation and functional maintenance require highly developed mitochondrial metabolism. In B cells derived from patients with SLE, constitutive activation of the PI3K-Akt-mTORC1 pathway downstream of BCR-Syk signaling has been reported, leading to sustained enhancement of mitochondrial activity (40). mTORC1 integrates anabolic metabolic programs, including mitochondrial biogenesis, oxidative phosphorylation, and amino acid utilization; this sustained activation may maintain B cells in a highly metabolic state suited for antibody production, rather than in a transient activation state. Consequently, metabolic abnormalities such as increased oxygen consumption rate, mitochondrial membrane hyperpolarization, and accumulation of ROS arise. These alterations have been shown to contribute to the formation of a pathogenic environment that promotes plasma cell differentiation (48). In addition, during the transition from activated B cells to germinal center B cells, mitochondrial transcription and translation, as well as mitochondrial remodeling mediated by transcription factor A, mitochondrial (TFAM), are essential. Throughout B-cell differentiation, mitochondria dynamically adjust their quantity and function and undergo continuous remodeling at each stage (53, 54). These findings suggest that mitochondrial activation is not merely a consequence of plasma cell differentiation, but rather serves a functional role in supporting the differentiation process itself. Furthermore, increased uptake of the essential amino acid methionine also influences plasma cell differentiation. Indeed, Zhang et al. demonstrated that, in the presence of methionine, activation of Syk and mTORC1 synergistically induces EZH2 expression; this leads to suppression of Bach2 and upregulation of BLIMP1 and XBP1, thereby promoting plasma cell differentiation (49). Taken together, these findings show that mTORC1 not only enhances metabolic activity but also promotes plasma cell differentiation by linking amino acid metabolism with chromatin modification.

In contrast, ABCs represent a B-cell subset that is characteristically expanded in SLE, displaying a distinctive phenotype marked by expression of the transcription factor T-bet and the myeloid marker CD11c, and are therefore also referred to as T-bet^+^CD11c^+^ B cells. Among the double-negative (DN) B cells (IgD^-^CD27^-^) that increase in peripheral blood, the DN2 subset (IgD^-^CD27^-^CD11c^+^T-bet^+^) corresponds to ABCs (9). Recent studies have demonstrated that ABCs are induced by IFN-γ stimulation and acquire a glycolysis-dependent metabolic program through T-bet-mediated suppression of Bcl6, leading to upregulation of glycolysis-related genes. In human B cells, inhibition of glycolysis has been shown to suppress the induction of differentiation into ABCs. Moreover, ABCs derived from patients with SLE exhibit increased expression of glycolytic enzymes (46). This enhanced glycolytic activity has been suggested to involve mTORC1 signaling. In immune cells, the mTORC1-glycolysis axis is considered a key metabolic foundation supporting inflammatory functions. A similar mechanism is also suggested to operate in B cells (27). Taken together, these findings indicate that enhanced glycolysis in ABCs is not merely involved in energy supply, but may also play a role in regulating their differentiation and inflammatory functions. This glycolysis-dominant metabolic profile is associated primarily with inflammatory cytokine production and antigen-presenting capacity rather than antibody production and is thought to contribute to SLE pathogenesis in a manner distinct from that of plasma cells.

In addition, SLE-specific metabolic reprogramming has also been reported in germinal center B (GCB) cells, which represent an intermediate stage of B-cell differentiation. Compared with T cells, activated B cells and GCB cells in lupus show higher dependence on glycolysis. Their survival is supported by metabolism that relies on glucose oxidation. In particular, these cells exhibit increased accumulation of mitochondrial reactive oxygen species (ROS). Moreover, inhibition of glycolysis selectively induces apoptosis in these cells. These findings suggest that glycolytic dependency represents a vulnerability of pathogenic B cells. Furthermore, a subset of GCB cells that express B-cell maturation antigen (BCMA) shows especially high glucose dependency. Elimination of these cells improves disease activity (55). Taken together, these findings suggest that increased glycolysis at the GCB stage potentially contributes to the selection and maintenance of autoreactive B cells.

In recent years, novel subpopulations within DN B cells, termed as DN3 B cells (IgD^-^CD27^-^CD11c^-^CXCR5^-^) and DN4 B cells (IgD^-^CD27^-^CD11c^+^CXCR5^+^), have been reported (56). These populations expand under chronic inflammatory conditions and may contribute to autoimmune disease pathogenesis, suggesting that they may represent important branching points in metabolic reprogramming. In particular, DN3 B cells have been reported to be increased in patients with SLE and to correlate with disease activity (57). In contrast, DN4 B cells have been described as a population enriched for IgE transcripts, but their functional significance and relevance to autoimmune diseases are yet to be clarified.

Furthermore, class-switched memory B cells (CD19^+^IgD^-^CD27^+^), conversely, exhibit excessive OXPHOS activation, which is closely associated with mitochondrial dysfunction. This metabolic reprogramming has been linked to differences in plasma cell differentiation (58). These findings suggest that aberrant B-cell differentiation in SLE is determined not merely by phenotypic differences but by distinct intracellular metabolic programs.

### Potential of metabolic markers for assessing SLE disease activity

Given the complex and heterogeneous pathogenesis of SLE, there is a need to establish biomarkers that can assist in its diagnosis, predict flares, and be used to monitor treatment response. Recent advances in metabolomic and gene expression analyses have facilitated the visualization of immunometabolic abnormalities, suggesting that metabolic alterations are closely associated with disease activity in SLE.

Metabolomic analyses have reported increased oxidative stress and elevated levels of cystine, a molecule involved in scavenging ROS, in the serum samples of patients with SLE (59). Furthermore, increased levels of glycoprotein acetyls, a marker of chronic inflammation, as well as elevated concentrations of glycolytic intermediates such as lactate and pyruvate, have been demonstrated (60). At the peripheral blood lymphocyte level, mTOR activation, glycolytic enzyme upregulation, increased oxidative stress, and higher cystine levels have also been observed, all of which are thought to reflect mitochondrial dysfunction (47, 61).

At present, gene expression analyses have not yet been implemented as practical biomarkers for directly assessing diagnosis or treatment responsiveness. Nevertheless, they provide important insights into the molecular basis of metabolic dysregulation and organ damage in SLE. Gene expression analyses using microarray technology have revealed reduced expression levels of genes related to the electron transport chain complex I in patients who progressed from antinuclear antibody (ANA) positivity to SLE, suggesting that mitochondrial dysfunction may be an early event in the development of autoimmune responses and organ damage (62). Gene expression profiling studies of renal tissue have also shown activation of OXPHOS and the pentose phosphate pathway, with the latter being particularly correlated with glomerular and tubulointerstitial injury, as well as with lower estimated glomerular filtration rates (63). These findings indicate that oxidative stress responses and metabolic reprogramming of energy production contribute to the progression of organ damage in patients with SLE.

Thus, metabolic reprogramming, including alterations in mitochondrial function, is consistently observed in the pathogenesis of SLE. These metabolic abnormalities are thought to reflect key aspects of the pathogenic basis underlying disease activity and organ damage. Nevertheless, these metabolic markers are not specific to B cells and should be interpreted with caution.

### New perspectives on SLE therapy through the regulation of B-cell metabolism

As intracellular B cell metabolism contributes to lupus pathogenesis, the regulation of glycolysis and OXPHOS has attracted particular attention as a potential therapeutic target for the disease.

The glycolysis inhibitor 2-deoxy-D-glucose (2-DG) has been reported to selectively suppress autoantibody production and ameliorate disease severity in lupus-prone mice (64). Similarly, the PKM2 inhibitor TEPP-46 reduced B cell–mediated inflammation by inhibiting glycolytic enhancement through Bcl6 signaling (65). Furthermore, glycolysis inhibitors have also been shown to decrease the proportion of ABCs (46). The glucose transporter inhibitor CG-5 and the CaMK4 inhibitor KN-93 have also been found to suppress the formation of germinal center B cells and autoantibody production, thereby contributing to the restoration of immune homeostasis (66, 67). Studies involving these mouse models suggest that inhibition of glycolysis contributes to the regulation of B-cell function and is associated with reduced autoantibody production and improved disease manifestations. However, therapeutic interventions targeting glycolysis are not restricted to B cells and may also affect multiple cell types, including T cells and innate immune cells. As a result, limited cell specificity and safety concerns associated with systemic metabolic suppression represent major challenges. In particular, because glucose metabolism is essential for many organs and tissues, careful evaluation of off-target effects and tolerability during long-term administration is required. In this regard, inhibitors of mTOR are also known to indirectly suppress glycolysis. In lupus-prone mice, their administration has been reported to reduce anti-dsDNA antibody levels, improve proteinuria, and increase survival (68). Sirolimus, an mTOR inhibitor, has recently demonstrated favorable tolerability and comparable efficacy to mycophenolate mofetil in patients with lupus nephritis (69). Therapeutic strategies targeting glycolysis have been shown to demonstrate some efficacy and safety in human studies.

The inhibition of mitochondrial metabolism is also considered an effective therapeutic strategy. Metformin inhibits mitochondrial complex I, leading to a reduction in intracellular ATP levels; this results in an increased AMP/ATP ratio, which activates AMPK and subsequently suppresses mTORC1 signaling (70, 71). Suppression of mTORC1 signaling reduces the expression of c-Myc-regulated glutamine transporters, thereby inhibiting glutamine uptake and suppressing plasma cell differentiation and antibody production (48). In murine models, combination therapy with metformin and 2-DG normalized T cell metabolism and inhibited mTORC1 activity, leading to the correction of metabolic abnormalities across multiple immune cell types (including B cells) and amelioration of lupus nephritis (72). In a clinical trial involving patients with SLE, metformin showed a trend toward reducing disease relapse, however, the difference relative to placebo was not statistically significant (73). This observation indicates that efficacy demonstrated in animal models is not necessarily directly translatable to human disease. Future efforts should focus on appropriate patient selection as well as optimization of dosing and treatment timing to advance clinical translation.

In recent years, therapeutic strategies targeting glutamine metabolism (glutaminolysis) have gained attention. DON-based inhibition of glutamine metabolism reduces autoantibody production and decreases germinal center B cells and ABCs. Furthermore, it has been suggested that B-cell responses are indirectly regulated through suppression of Tfh cell function, including reduced ICOS expression (74). Furthermore, the GLS1 inhibitor CB839 decreases Tfh cells, activates B cells in lupus-prone mice, and improves disease activity by suppressing mTOR signaling and inflammatory pathways (75). These findings suggest that glutamine metabolism is closely associated with immune cell function and contributes to the regulation of autoimmune disease pathogenesis.

Thus, therapeutic interventions targeting metabolic reprogramming are expected to represent a novel treatment strategy for SLE, distinct from conventional immunosuppressive approaches. However, many of these therapeutic approaches currently lack selectivity for specific immune cell types. As a result, from the perspective of B-cell specificity, their efficacy and safety remain insufficiently validated. Recently, drug delivery strategies targeting cell-specific surface molecules have attracted attention as an approach to overcome this limitation. In particular, methods in which antibodies against immune checkpoint molecules or cell surface antigens are conjugated to the surface of nanoparticles, with inhibitors of the PI3K-AKT-mTOR pathway encapsulated within the nanoparticles for delivery into target cells, have been reported. Such approaches have demonstrated effective antitumor activity in mouse models in the field of cancer immunotherapy (76). By applying these technologies, B-cell-specific metabolic regulation could be achieved and more precise and novel therapeutic strategies can be established in the future.

## Conclusion

The metabolic reprogramming of glycolysis and mitochondrial metabolism plays a crucial role in SLE pathogenesis, contributing to excessive autoantibody production and increased inflammatory cytokine secretion. In SLE, B cells acquire distinct metabolic programs depending on their stage of differentiation. Differences in these metabolic features determine B-cell activation, differentiation fate, and the manner in which B cells contribute to disease pathogenesis. If these metabolic differences are indeed involved in determining the fate of B cell differentiation, this represents an intriguing mechanism linking metabolism to immune regulation. Such insights may deepen our understanding of SLE pathogenesis and contribute to the development of novel therapeutic strategies that complement existing therapies.
